# Validation and Application of Biocrates Absolute*IDQ*^®^ p180 Targeted Metabolomics Kit Using Human Milk

**DOI:** 10.3390/nu11081733

**Published:** 2019-07-26

**Authors:** Daniela Hampel, Setareh Shahab-Ferdows, Muttaquina Hossain, M. Munirul Islam, Tahmeed Ahmed, Lindsay H. Allen

**Affiliations:** 1 USDA/ARS Western Human Nutrition Research Center, 430 West Health Sciences Drive, Davis, CA 95616, USA; 2 Department of Nutrition, University of California, One Shields Ave, Davis, CA 95616, USA; 3 Nutrition and Clinical Services Division, International Centre for Diarrhoeal Disease Research, 68 Shaheed Tajuddin Ahmed Sarani, Mohakhali, Dhaka 1212, Bangladesh

**Keywords:** human milk, targeted metabolomics, amino acids, lipid metabolites, LC-MS, flow injection analysis

## Abstract

Human-milk-targeted metabolomics analysis offers novel insights into milk composition and relationships with maternal and infant phenotypes and nutritional status. The Biocrates Absolute*IDQ*^®^ p180 kit, targeting 40 acylcarnitines, 42 amino acids/biogenic amines, 91 phospholipids, 15 sphingolipids, and sum of hexoses, was evaluated for human milk using the AB Sciex 5500 QTRAP mass-spectrometer in liquid chromatography-tandem mass-spectrometry (LC-MS/MS) and flow-injection analysis (FIA) mode. Milk (<6 months lactation) from (A) Bangladeshi apparently healthy mothers (body mass index (BMI) > 18.5; n = 12) and (B) Bangladeshi mothers of stunted infants (height-for-age Z (HAZ)-score <−2; n = 13) was analyzed. Overall, 123 of the possible 188 metabolites were detected in milk. New internal standards and adjusted calibrator levels were used for improved precision and concentration ranges for milk metabolites. Recoveries ranged between 43% and 120% (coefficient of variation (CV): 2.4%–24.1%, 6 replicates). Milk consumed by stunted infants vs. that from mothers with BMI > 18.5 was lower in 6 amino acids/biogenic amines but higher in isovalerylcarnitine, two phospholipids, and one sphingomyelin (*p* < 0.05 for all). Associations between milk metabolites differed between groups. The Absolute*IDQ*^®^ p180 kit is a rapid analysis tool suitable for human milk analysis and reduces analytical bias by allowing the same technique for different specimens. More research is needed to examine milk metabolite relationships with maternal and infant phenotypes.

## 1. Introduction

The postnatal period is a critical stage for infant’s physiology, accompanied by rapid changes in brain development, and in metabolic, immunologic, intestinal, and physiological systems [1]. Human milk undergoes dynamic changes in composition during lactation to provide all nutrients and other bioactive substrates for optimal infant growth and development, and exclusive breastfeeding (EBF) is recommended during the first 6 months of life [2]. Even beyond the EBF period, it remains a vital dietary source of nutrients; however, the mechanisms behind its ability to drive and affect infant metabolism, health, development, and long-term outcomes are not fully understood. The recent resurgent interest in human milk composition triggered the development of state-of-the-art methods for analyzing micronutrients [3,4,5,6,7,8,9,10], peptides and oligosaccharides (HMOs) [11,12], the human milk microbiome [13,14], and milk lipids [15,16]. Within this scope, metabolomics provides novel insight into the complexity of the milk metabolome and its influence on infant metabolism and physiology via intestinal enzymes [17,18].

The effects of human milk bioactives on early infant physiology and development are diverse; effects may reach the insulin signaling cascade, the hepatic mitochondrial system, and possibly the mechanistic target of rapamycin (mTOR) signaling pathway, vital to cell growth, protein and lipid synthesis, or lipid and adiposity accumulation and adipogenesis [1,19,20,21,22]. On the other hand, associations of human milk metabolites were also found with maternal phenotype and diet [22,23]. The observed relationships of milk metabolites and maternal and infant status and phenotypes demand further investigation and analyzing the human milk metabolome as the interface between mother and infant can provide key information for maternal and infant health. Moreover, milk metabolites are not only derived from maternal blood but also from *de novo* synthesis in the mammary gland [23,24]. Consequently, obtaining the metabolic profile of the mothers as well as their milk may offer important insights into the contributions of the maternal metabolic state to the milk metabolome compared to the mammary-gland-derived compounds. Using the same analytical technique on different matrices reduces method bias and produces more robust results.

The Absolute*IDQ*^®^ p180 kit (https://www.biocrates.com/images/p180_Folder_HP_v01-2018.pdf) from Biocrates Life Science AG covers multiple compound classes (acylcarnitines, amino acids, biogenic amines, sphingolipids, phospholipids, and sum of hexoses) which are involved in various central metabolic processes, such as immune regulation, fatty acid oxidation, membrane composition, cell cycle control, insulin resistance, or nutritional status. While this assay has been validated for various species and matrices, the human milk matrix has not yet been evaluated. Therefore, the purpose of this study was to assess application of this kit for the analysis of human milk metabolites. The validated assay was then used in a feasibility plate to analyze milk samples from (A) apparently healthy Bangladeshi mothers (“BMI > 18.5 mothers” (BMI—body mass index), < 6 months lactation) and (B) mothers of stunted infants (“stunted infants”, height-for-age Z (HAZ)-score <−2) as proof of concept.

## 2. Materials and Methods

### 2.1. Chemicals and Materials

All reagents, internal and calibration standards, quality controls, test mix, and a patented 96-well filter plate required for the Absolute*IDQ*^®^p180 analysis were included in the kit or provided by Biocrates Life Science AG (Innsbruck, Austria).

### 2.2. AbsoluteIDQ^®^ p180 Assay and Sample Preparation

The Absolute*IDQ*^®^ p180 kit is a fully automated assay based on phenylisothiocyanate (PITC) derivatization of the target analytes in bodily fluids (e.g., plasma, serum, urine) using internal standards for quantitation. Amino acids and biogenic amines are determined in LC-MS mode, acylcarnitines, phospholipids (lyso-phosphatidylcholines with acyl residue at CXX:X, phosphatidylcholine with diacyl residue sum CXX:X (PC aa), and phosphatidylcholine with acyl-alkyl residue sum CXX:X (PC ae)), sphingomyelins, and the sum of hexoses are analyzed in flow injection analysis (FIA). Human milk sample preparation was carried out according to the manufacturer’s protocol. Briefly, 2 to 10 µL of human milk was transferred to the upper 96-well plate and dried under a nitrogen stream. Thereafter, 50 µL of a 5% PITC solution was added to derivatize amino acids and biogenic amines. After incubation, the filter spots were dried again before the metabolites were extracted using 5 mM ammonium acetate in methanol (300 µL) into the lower 96-well plate for analysis after further dilution using the MS running solvent A. Quantification was carried out using internal standards and a calibration curve (Cal 1 to Cal 7). The experimental metabolomics measurement technique is described in detail by patents EP1897014B1 and EP1875401B1 [25,26]. See Supplementary Materials (Table S1) for the full list of metabolites and their abbreviations.

### 2.3. LC-MS

The LC-MS/MS system was comprised of an ACQUITY UPLC-system (Waters, Milford, MA, USA) coupled to a QTRAP 5500 mass spectrometer (AB Sciex, Redwood City, CA, USA) in electrospray ionization (ESI) mode. Amino acids and biogenic amines were analyzed via LC-MS in positive mode. Two microliters of the sample extract were injected onto an ACQUITY UPLC BEH C18 column, 2.1 × 7.5 mm, 1.7 µm protected by an ACQUITY BEH C18, 1.7 µm VanGuard pre-column (Waters, Milford, MA, USA) at 50 °C using a 7.3 min solvent gradient employing 0.2% formic acid in water (solvent A) and 0.2% formic acid in acetonitrile (solvent B).

Twenty microliters of the sample extract were used in the flow injection analysis (FIA) in positive mode to capture acylcarnitines, glycerophospholipids, and sphingolipids, while hexoses were monitored in a subsequent run in negative mode. All FIA injections were carried out using the Biocrates MS Running Solvent. Additional LC and MS settings for LC-MS and FIA mode are described in Table 1. All metabolites were identified and quantified using isotopically-labeled internal standards and multiple reaction monitoring (MRM) as optimized and provided by Biocrates Life Sciences AG (Innsbruck, Austria).

### 2.4. Method Validation

Pooled milk was used to evaluate matrix effects and metabolite recovery. The samples were diluted in phosphate buffered saline solution (PBS) 1:2 and 1:5 to assess ion suppression due to the matrix. A 3-level analyte recovery was carried out using Cal 2, 3, and 5 in half, normal, and double concentrations, covering up to 230-fold of the endogenous milk concentrations. All experiments were conducted in replicates of six. Individual milk samples (n = 25) from Bangladeshi women were prepared according to the manufacturer’s protocol and analyzed at 3 different sample volumes (2, 5, and 10 µL).

Analyte recovery was calculated using the following equation:

Recovery [%] = (C_measured_—C_endogenous_) × 100/C_added_ (1)

where C_measured_ is the measured concentrations of the spiked sample, C_endogenous_ the measured concentration of the non-spiked sample, and C_added_ the theoretically added concentration by spiking.

Relative recovery in the diluted samples was calculated as follows:

Relative recovery [%] = C_X/dF_ × dF × 100/C_X_ (2)

where C_X/dF_ is the concentration measured in the diluted sample, dF the dilution factor (2 or 5), and C_X_ the concentrations measured in the non-diluted milk samples.

### 2.5. Human Milk Samples

To determine recovery rates and evaluate matrix effects, a human milk pool, obtained from milk samples provided by apparently healthy women in the Vancouver, BC, Canada area, was used. Individual milk samples, available in the laboratory but not collected for the purpose of this validation, were analyzed in the feasibility plate. These convenient samples were collected during the first 6 months of lactation from apparently healthy Bangladeshi lactating women (“BMI > 18.5 mothers”, body mass index (BMI) > 18.5, n = 12) whose infants’ anthropometry was unknown, and Bangladeshi mothers with stunted infants (“stunted infants”, HAZ-score < −2, n = 13), to try to obtain a wide range of metabolite concentrations in the milk. All milk samples were shipped from the United States Department of Agriculture – Agricultural Research Service (USDA/ARS) Western Human Nutrition Research Center in Davis, CA, USA on dry ice to Biocrates Life Science AG in Innsbruck, Austria for testing. Upon arrival, samples were stored at −80 °C until analysis.

### 2.6. Statistical Analysis

Raw data was computed in Met*IDQ*^TM^ version Carbon (Biocrates Life Science AG, Innsbruck, Austria). Mean, standard deviation, and coefficient of variation (CV) for the validation were calculated in Excel 2016 (Microsoft Corporation, Redmond, WA, USA). R statistical software (3.5.2, R Foundation for Statistical Computing) was used for statistical analysis and visualization of the results for the feasibility study. The Wilcoxon rank sum test was used to explore differences in analyte concentrations based on group assignment (BMI > 18.5 mothers vs. stunted infants), and metabolite associations were assessed using Spearman’s rank correlation. *p*-values < 0.05 were considered significant.

## 3. Results

### 3.1. Validation Using Pooled Human Milk

#### 3.1.1. LC-MS (Amino Acids and Biogenic Amines)

Since the development of the assay, new internal standards became available for lysine, acetyl-ornithine, alpha-aminoadipic acid, cis-4-OH-proline, histamine, kynurenine, nitro-tyrosine, phenylethylamine (PEA), symmetric dimethylarginine (SDMA), and trans-4-OH-proline. Using these new internal standards, mean CVs (range) were reduced from 11.3% (3.6%–26.6%) to 6.6% (2.3%–11.5%; *p* = 0.017, Student’s paired t-test). The quantification of glutamate and taurine required additional calibrators (Cal 8 and 9), which was also true for putrescine (Cal 8). Saturation effects were observed for proline, tryptophan, and valine at Cal 5 and Cal 6 levels. The sensitivity of the 5500 QTRAP MS-system allowed the extension to calibrators (Cal 0.5 and 0.25) for most of the metabolites and was needed for asparagine, methionine, ornithine, tryptophan, asymmetric dimethylarginine (ADMA), kynurenine, methionine-sulfoxide, sarcosine, and SDMA. However, alanine, spermidine, spermine, and ADMA could not be accurately measured at the Cal 0.25 level.

**Figure 1:**
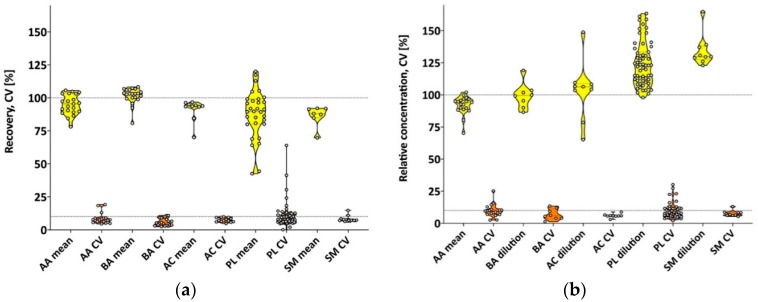
(**a**) Violin plots of mean milk metabolite recovery and coefficients of variation (CVs) determined by 3-level spiking experiments (n = 6); (**b**) relative milk metabolite recovery and CVs of the diluted milk samples. AA—amino acids; BA—biogenic amines; AC—acylcarnitines; PL—phospholipids; SM—sphingomyelins.

All 21 amino acids and 12 biogenic amines were detectable in the pooled human milk sample. Overall recovery across three spiking levels ranged between 79%–106% (CV: 4.5%–19.2%) for amino acids, and 81%–108% (CV: 2.4%–11.0%) for biogenic amines, as shown in Figure 1a and Supplementary Table S2. Compared to the non-diluted milk samples, the diluted pooled milk revealed a relative concentration range for amino acids of (1:2/1:5 dilution) 70%–100%/67%–104% (CV: 2.4%–15.7%/2.7%–11.0%) and 87%–119%/80%–102% (CV: 0.8%–13.5%/10.8%–14.6%) for biogenic amines, as shown in Figure 1b and Supplementary Table S3. Ornithine relative concentration was in good agreement with the non-diluted milk samples for the 1:2 dilutions (96.7%) but revealed a higher variation of CV = 25.3%. However, some of the amino acids and biogenic amines, including ornithine, were only observed in very low concentrations in the non-diluted pooled milk samples.

#### 3.1.2. FIA (Acylcarnitines, Phospholipids, Sphingomyelins, Hexoses)

Sample extracts were diluted 1:20 for analysis using the 5500 QTRAP mass spectrometer. A relatively low recovery (CV) for PC aa C32:1 (42.6% (41.3%)) and to a lesser extent for PC aa C42:1 (44.4% (15.0%)) were observed. Overall, 89 (11 acylcarnitines, 63 phospholipids, 15 sphingomyelins) out of 146 FIA-analytes were quantifiable, with five additional metabolites detectable above (LOD) with no measurable carry-over effects. Overall recovery across the three spiking levels ranged between 70%–97% (CV: 5.8%–9.6%; acylcarnitines), 70%–92% (CV: 6.4%–8.6%; sphingomyelins), and 91.4% (CV: 7.1%) for the sum of hexoses. Excluding the above mentioned phospholipids, the recovery ranges between 64%–120% (CV: 3.9%–15.9%). PC ae C30:1 showed a good recovery of 85.7% but high variation (CV: 24.1%), as shown in Figure 1a and Supplementary Table S4.

Compared to the concentrations measured in the non-diluted pooled milk samples, acylcarnitines revealed relative concentration ranges (1:2/1:5 dilution) of 70%–134%/60%–164% (CV: 3.0%–7.0%/6.7%–10.7%), sphingomyelins ranged between 110%–167%/135%–228% (CV: 4.1%–13.0%/6.1%–20.5%), and hexoses 111%/143% (CV: 6.0%/10.5%). Phospholipids relative concentrations spanned between 97%–158%/95%–173% (CV: 1.8%–19.3%/2.3%–28.5%). Only five of the metabolites had a CV > 20% at the higher dilution. PC aa C32:1 and PC ae C30:1 experienced higher variations (CVs: 27% to 36.3%). PC aa C 38:1 was not detectable at a higher dilution and showed extreme variations at the lower dilution level with a mean relative concentration of 157% and CV = 55.7%, as shown in Figure 1b and Supplementary Table S5.

### 3.2. Feasibility Plate

Across all analyzed milk samples (BMI > 18.5 mothers and stunted infants), all 21 amino acids, 15 sphingomyelins, and the sum of hexoses were quantifiable. In addition, 10 biogenic amines, 11 acylcarnitines, and 54 phospholipids were observed, as shown in Supplementary Table S6.

Milk from BMI > 18.5 mothers had significantly higher concentrations of the amino acids and biogenic amines citrulline, glutamate, glycine, phenylalanine, serine, and the biogenic amine sarcosine (all *p* < 0.045), and lower concentrations of isovalerylcarnitine, phosphatidylcholine with diacyl residue sum C36:6 (PC aa), phosphatidylcholine with acyl-alkyl residue sum C30:2 (PC ae), and sphingomyelin with acyl residue sum C22:3 (all *p* < 0.048), as shown in Table 2, when compared to milk fed to stunted infants.

**Figure 2:**
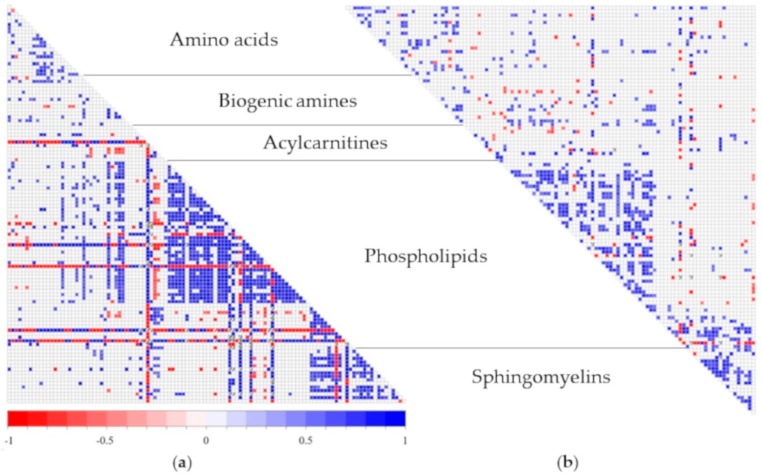
Spearman rank correlation map of milk metabolites in (**a**) BMI > 18.5 mothers and (**b**) stunted infants groups. Only significant correlations are shown (*p <* 0.05).

While milk metabolites were significantly associated (*p <* 0.05) in both groups, the correlation profile differed considerably. These significant relationships were found not only among metabolites that were significantly different in concentration between the two groups but also among metabolites that had similar concentrations in all milk samples across groups, as shown in Figure 2.

## 4. Discussion

### 4.1. Validation Using Pooled Human Milk

LC-MS: The new internal standards for amino acids and biogenic amines, and the extension of the calibrator curve allowed a more accurate analysis of the milk metabolites. While proline, tryptophan, and valine revealed saturation effects at mid calibrator levels, the measured milk concentrations of these amino acids, however, were well within the linear calibrator curve and the high level calibrators were not needed. While the low range of the calibrator curve was also extended to Cal 0.5 and 0.25, alanine, spermidine, spermine, and ADMA were not quantifiable at the lowest calibrator. Given that these metabolites were present in milk above Cal 0.5, the low accuracy at Cal 0.25 concentrations was not problematic. Some of the amino acids and biogenic amines, including ornithine, were found already in very low concentrations in the non-diluted pooled milk sample; consequently, their concentrations in the diluted samples were around or below the limit of detection (LOD) and reliable data could not be obtained. For metabolites within the calibrator curve, the diluted samples were mostly within 80%–120%, indicating some but not serious effects for quantification due to the matrix. The lower ion spray voltage (IS) intensity found for milk samples when compared to plasma further confirmed effects due to the milk matrix. To reduce matrix interferences due to early eluting matrix components (e.g., lactose), results for creatinine, the first eluting metabolite in LC-MS mode could be sacrificed to maintain MS-integrity and accuracy over time.

FIA: Phospholipids showed the greatest variation in metabolite recovery, and also represented the largest compound class of metabolites analyzed. Some of the variation could be explained by low abundance of metabolites at the edges of the calibrator range, or standard additions possibly to levels of saturation and therefore loss of linearity and accuracy. However, selectivity issues by milk-specific interferences cannot be excluded. No extended calibrator curve was necessary in FIA mode. The relative concentration of the analytes in the diluted samples compared to the non-diluted samples of generally above 100%, and often above 150% at higher dilutions, suggested some ion suppression due to the matrix which can be overcome by sample dilution. However, no metabolite was affected by the matrix to a degree that would not allow quantitation. However, given the abundance of lactose in the sample (~7%), a greater dilution would be preferable to reduce contamination of the MS by matrix constituents in FIA mode.

We found all detectable amino acids, sphingomyelins, and the sum of hexoses in human milk, but only 25% of the acylcarnitines, 50% of the biogenic amines, and 68% of the phospholipids. A recent study on plasma metabolites of healthy men and women (18–80 years) using the Absolute*IDQ*^®^ p180 kit reported fewer of the amino acids, biogenic amines, acylcarnitines, and sphingomyelins in plasma, but more phospholipids [27], illustrating the variations in metabolite profile depending on specimen analyzed. Moreover, that study reported that the metabolic profile of plasma and urine can predict sex with >90% accuracy, as well as age (based on gender), and within females the menopausal status could be predicted with >80% accuracy. Other studies showed that differences in plasma metabolite profiles could be a useful diagnostic tool for Alzheimer’s disease and cognitive impairment, or type 2 diabetes [28,29], further indicating the importance and relevance of the information than can be derived from the milk metabolome to the phenotype and status [27].

### 4.2. Feasibility Plate

#### Differences by Group Assignment

Overall, the metabolites detected in the feasibility plate using milk from Bangladeshi women were similar to those in the pooled human milk sample. The observed higher concentrations in milk from BMI > 18.5 mothers for selected amino amides and biogenic amines and lower concentrations of phospholipids, acylcarnitines, and sphingomyelins are consistent with alterations found in the serum metabolome of stunted compared to non-stunted children (1 to 5 years of age) in Malawi by Semba et al., using the same Absolute*IDQ*^®^ p180 kit [30]. Stunting can be caused by intrauterine growth retardation, inadequate nutrition for optimal infant growth and development, or repeated infections early in life, and usually originates in utero [31]. Thus, the reported low circulating amino acids and other metabolome alterations in the blood of stunted children in Malawi may stem from challenges in utero and early infancy, including possible effects derived from the lower supply of amino acids in human milk as reported here. This hypothesis is supported by the fact that human growth is regulated by the mechanistic target of rapamycin complex C1 (mTORC1), a major growth regulating pathway, which suppresses protein and lipid synthesis and cellular growth when intake of specific amino acids is insufficient [30,32]. However, since we do not have the full information about maternal and infant characteristics, including exact stage of lactation for each sample, we cannot exclude that additional maternal or infant factors possibly affect the milk metabolome. Nonetheless, correlation patterns changed in milk fed to stunted infants, indicating alterations of the milk metabolome and its pattern could be related to the infant phenotype. Since the alterations in the metabolome correlation profile were not limited to metabolites showing significant differences in concentrations, these relationships may be also affected by maternal or infant status. However, our sample size was very small, the anthropometric status of infants in the group of BMI > 18.5 mothers was unknown, and further research is needed to confirm and explore the results found in this feasibility study.

## 5. Conclusions

This is the first study to validate the commercially available Absolute*IDQ*^®^ p180 assay for targeted metabolomics in the human milk matrix. A greater range of calibrators is needed to accommodate the wide range of milk metabolite concentrations; new available internal standards improved accuracy of the results. While the primary purpose of this study was the validation of the Absolute*IDQ*^®^ p180 assay for the human milk matrix, and it was not our intention to draw conclusions about group differences in metabolites from the feasibility plate, the observed differences in concentrations and metabolome profiles in the two groups warrant further investigation of the mother–infant relationship and the importance of milk metabolome for infant optimal growth and development, which is now possible using identical targeted metabolomics assays for blood and milk samples.

## Figures and Tables

**Table 1:** LC and MS parameters ^1^.

Instrument	Parameter	LC-MS	FIA (MS Only)
5500 QTRAP MS	CUR	45	20
	IS	5500	5500
	TEM	500	200
	GS1	60	40
	GS2	70	50
	CAD	9	9
	EP	10	10
	CXP	15	15
ACQUITY UPLC	**Time [min]**	**Flow rate [mL/min]**	**Solvent A [%]**
LC-MS	0	0.8	100
	0.45	0.8	100
	3.3	0.8	85
	5.9	0.8	30
	6.05	0.8	0
	6.2	0.9	0
	6.42	0.9	0
	6.52	0.8	0
	6.7	0.8	100
	7.3	0.8	100
FIA	0	0.03	Biocrates Solvent I MS running buffer in isocratic mode
	1.6	0.03	
	2.4	0.2	
	2.8	0.2	
	3.0	0.03	

^1^ CUR—curtain gas; IS—ion spray voltage (V);—temperature (°C); GS1/GS2—ion source gas 1 and 2 (psi); CAD—CAD gas (psi); EP—entrance potential (V); CXP—collision cell exit potential (V); LC-MS—liquid chromatography—mass spectrometry; FIA—flow injection analysis.

**Table 2:** Median concentrations and interquartile range (IQR) for milk metabolites affected by group (BMI > 18.5 mothers vs. stunted infants).

Metabolite ^1^	BMI > 18.5 Mothers	Stunted Infants	*p*-Value ^2^
	[µmol/L]		
C 5	0.13 (0.12, 0.14)	0.19 (0.17, 0.37)	0.011
PC aa C36:6	0.028 (0.025, 0.028)	0.031 (0.028, 0.035)	0.044
PC ae C30:2	0.011 (0.01, 0.011)	0.012 (0.012, 0.013)	0.047
SM C22:3	0.017 (0.014, 0.019)	0.022 (0.018, 0.037)	0.014
Citrulline	18.5 (12.2, 22.3)	10.6 (8.7, 16.3)	0.021
Glutamate	1825 (1477, 1977)	1181 (976, 1472)	0.002
Glycine	116 (103, 147)	96.0 (79.5, 104)	0.017
Phenylalanine	12.5 (11.7, 16.1)	11.2 (10.3, 12.0)	0.012
Serine	142 (94.2, 184)	85.0 (79.4, 108)	0.026
Sarcosine	0.61 (0.52, 0.72)	0.46 (0.38, 0.51)	0.044

^1^ C 5—isovalerylcarnitine/2-methylbutyrylcarnitine/valerylcarnitine; PC aa C36:6—phosphatidylcholine with diacyl residue sum C36:6; PC ae C30:2—phosphatidylcholine with acyl-alkyl residue sum C30:2; SM C22:3—sphingomyelin with acyl residue sum C22:3. ^2^ *p*-values by Wilcoxon rank sum test.

## Supplementary Materials

The following are available online at https://www.mdpi.com/2072-6643/11/8/1733/s1, Table S1: List and abbreviations of metabolites, Table S2: Metabolite concentrations (mean ± SD) and recovery rates (CV) of pooled milk in LC-MS mode, Table S3: Relative recovery (mean ± SD) of milk metabolites in diluted pooled human milk based on non-diluted samples in LC-MS mode, Table S4: Metabolite concentrations (mean ± SD) and recovery rates (CV) of pooled milk in FIA mode, Table S5: Relative recovery (mean ± SD) of milk metabolites in diluted pooled human milk based on non-diluted samples in FIA mode, Table S6: Median concentrations and interquartile ranges (IQR) of milk metabolites in the BMI > 18.5 mothers and the stunted infants groups.
